# COVID-19 and Recreational Skiing: Results of a Rapid Systematic Review and Possible Preventive Measures

**DOI:** 10.3390/ijerph18084349

**Published:** 2021-04-20

**Authors:** Vincenza Gianfredi, Nicole Sibilla Mauer, Leandro Gentile, Matteo Riccò, Anna Odone, Carlo Signorelli

**Affiliations:** 1 School of Medicine, University Vita-Salute San Raffaele, 20132 Milan, Italy; gianfredi.vincenza@hsr.it (V.G.); mauer.nicolesibilla@hsr.it (N.S.M.); signorelli.carlo@hsr.it (C.S.); 2 CAPHRI Care and Public Health Research Institute, Maastricht University, 6211 Maastricht, The Netherlands; 3 Department of Public Health, Experimental and Forensic Medicine, University of Pavia, 27100 Pavia, Italy; leandro.gentile01@universitadipavia.it (L.G.); anna.odone@unipv.it (A.O.); 4 Medical Direction, Fondazione IRCCS Policlinico San Matteo, 27100 Pavia, Italy; 5 AUSL-IRCCS di Reggio Emilia, Servizio di Prevenzione e Sicurezza negli Ambienti di Lavoro (SPSAL), Via Amendola n.2, I-42122 Reggio Emilia, Italy

**Keywords:** SARS-CoV-2, COVID-19, systematic review, ski, skiing

## Abstract

COVID-19 is a novel infectious disease which has rapidly spread around the globe, disrupting several aspects of public life over the past year. After numerous infection clusters emerged among travelers hosted in ski resorts in early 2020, several European countries closed ski areas. These measures were mostly upheld throughout the 2020 and 2021 winter season, generating significant economic loss for mountain communities. The aim of this rapid systematic review was to explore the association between recreational skiing and the spread of COVID-19. This review was conducted according to the WHO practical guidelines on rapid reviews and the Preferred Reporting Items for Systematic Reviews and Meta-Analyses guidelines. PubMed, Scopus, MedRxiv and Promed-mail were screened to identify relevant scientific and grey literature published since the emergence of COVID-19. Among the 11 articles included, seven focused on cases recorded during the first epidemic wave, when COVID-19 containment measures were not yet mandatory. Most infection clusters could be directly linked to public gatherings which took place without the enforcement of restrictions. There is currently no evidence to suggest an association between COVID-19 spread and recreational skiing. It may be reasonable to consider the reopening of ski areas in compliance with strict rules and preventive measures.

## 1. Introduction

SARS-CoV-2 is a novel highly infectious coronavirus first detected in the region of Wuhan (China) at the end of 2019 [1]. Over a short period of time, the infection spread globally [2], culminating in the declaration of a pandemic by the World Health Organization (WHO) in March 2020 [3]. The virus mainly transmits between humans through respiratory droplets from the upper respiratory tract causing an acute respiratory syndrome with potentially severe symptoms and outcomes. Due to the novelty of the disease, lack of specific preventive measures (such as vaccines, which only became widely available in January 2021) and effective treatment options, many unspecific preventive measures (i.e., nonpharmaceutical interventions, NPI) were put in place globally to limit disease transmission, including social distancing, stricter guidelines on hand hygiene, the compulsory use of face masks [4], travel restrictions [5], lockdown measures [6,7], contact tracing [8], quarantine and home isolation [9], the targeted use of new health technologies [10] and an adequate risk communication [11,12]. The implementation of such measures has pervaded all aspects of daily life, resulting in school closures, work from home and limitations to recreational activities including skiing.

Recreational skiing and all facilities involved, including departure stations with ski pass sale points, ski lifts, refuges and chalets, ski schools and rental shops, have been considered to be at high-risk of facilitating infection spread throughout the SARS-CoV-2 pandemic. Particularly as new viral variants with greater transmission potential are starting to emerge and circulate. During the first epidemic wave (February–May 2020) [13,14], several international clusters originated from infected travelers who were hosted in ski areas. For this reason, and with the intention to reduce the spread of SARS-CoV-2, several European governments decided to close ski areas. In the majority of cases, closure of ski areas was also confirmed during the 2020 and 2021 winter season as a preventive measure. However, this decision has carried numerous social and economic implications, particularly for those mountain communities dependent on skiing facilities to secure their financial existence. Based on these considerations and careful monitoring of the epidemic curve, Switzerland maintained ski areas open throughout the winter season (during the second and third epidemic wave—November 2020–February 2021), whereas neighboring countries such as Austria, Germany, France and Italy implemented substantial restrictions and even the complete closure of facilities over the same period. Although skiing is an individual outdoor sport, it is a suspected amplifier of infection due to the potential for crowding and queueing at ticket offices, ski lifts, restaurants or cafés, public transportation and shared areas as in hotels or chalets. Despite the approval of a precautionary protocol by the government’s technical scientific committee (CTS) recommending measures aimed at limiting the risk of contagion in ski areas on the 15 February 2021, the Italian Ministry of Health postponed the reopening of ski areas for the third consecutive time, a last-minute decision which fueled dissatisfaction among workers in the sector, highlighting the importance of an advocacy process between policymakers and public health professionals [15].

With the aim of evaluating the available scientific evidence on the risks of recreational skiing for the evolution of the COVID-19 pandemic, we carried out a rapid systematic review consulting both scientific and grey literature sources.

## 2. Materials and Methods

We performed a rapid systematic review conducted according to the World Health Organization (WHO) guidelines: “Rapid reviews to strengthen health policy and systems: a practical guide” [16] and reported following the Preferred Reporting Items for Systematic Reviews and Meta-Analyses (PRISMA) guidelines [17]. In the digital era and in the context of a rapidly evolving situation, rapid reviews are an extremely useful new instrument for collecting and synthetizing emerging evidence that can be easily and promptly used by policymakers to inform their decisions. Policymakers need a solid and up-to-date evidence base to support the implementation and development of effective health policies. This is particularly true when difficult decisions have to be made in circumstances of emergency, such as the current COVID-19 pandemic.

We used PubMed/Medline and Scopus to screen the scientific literature. In order to identify eligible documents from the grey literature, we consulted the medRxiv platform for preprints and ProMed-mail, a digital infectious disease surveillance tool. Additionally, we searched the webpages of international health authorities (including the WHO, the Centers for Disease Control and Prevention—CDC, and the European Centre for Disease Prevention and Control—ECDC) and consulted experts in the field. Reference lists of included articles were also screened in order to identify any potentially relevant articles. The literature search was carried out on the 15 February 2021, combining the key words “ski” or “skiing” and “COVID-19” or “SARS-CoV-2”, both using free text words and medical subject headings (MeSH). No time filter was applied, but only original articles published in English, addressing the risk of SARS-CoV-2 spread linked to skiing activities, were considered eligible. We subsequently updated the search on the 1 April 2021. Articles were first assessed based on title and abstract and only eligible papers were evaluated in full. Data extraction was performed using a pre-piloted spreadsheet elaborated in Microsoft Excel^®^ for Windows (Microsoft Corporation, Redmond, WA, USA).

## 3. Results

### 3.1. Literature Search

**Figure 1:**
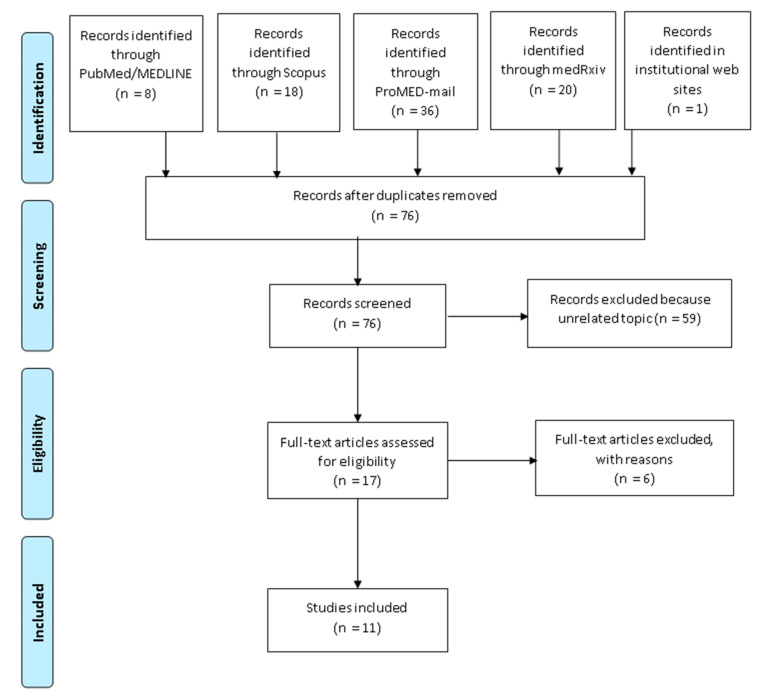
Flow diagram depicting the selection process.

A total of 38 articles were retrieved, of which 8 in PubMed/MEDLINE, 18 in Scopus, and 20 preprints from the platform medRxiv; however, seven articles were duplicates and were immediately removed. In ProMed-mail, six updates including a total of 36 posts were retrieved, of which only two met inclusion criteria [18,19]. Scanning the webpages of Health Authorities, only one relevant report from the ECDC was detected [20]. Hence, a total of 76 articles and posts were screened, and based on title and abstract, a total of 59 were excluded because considered irrelevant. A total of 17 articles were assessed in full, and 6 were excluded due to the following reasons: Four articles reported on cases which originated in ski areas but did not add any further information or details [21,22,23,24]. Another article discussed an infectious disease outbreak management tool for endurance mass-participation sporting events [25]. Finally, there was an article considering the consequences of confinement among athletes [26]. At the end of the selection process, a total of 11 articles were included in the review [18,19,20,27,28,29,30,31]. Figure 1 illustrates the selection process.

### 3.2. Characteristics of Included Studies

Among the 11 retrieved articles, nine were scientific articles [18,27,28,29,30,31], of which one in press [18]; the other two articles included an institutional report [20] and a newspaper article [19]. The remaining two articles were preprints [32,33]. Among the included studies, those that demonstrated a potential role of ski areas in the spread of COVID-19 (n = 7), referred only to the first epidemic wave (February–March 2020) [27,28,29,30,31]. The majority of collected evidence was based on reports from Western Europe, with only one preprint by McLaughlin et al. [33] addressing the pandemic in North America. More specifically, these articles report how, through contact tracing and diagnostic tests, it was possible to reconstruct the chain of contagion of some international outbreaks that occurred through index cases that had previously stayed in ski resorts. For instance, an index case in France, Contamines-Monjioie, initiated clusters in Spain (Mallorca) and the United Kingdom (London) [30], whereas another index case in Austria (Ischgl) was linked to clusters in Germany, Norway, Denmark and Iceland [29]. In another study, where the index case was located in the French Alps, infection spread to Marseille [27]. However, only in some of these cases it was possible to document a transmission route. Notably, the circumstances of disease transmission were not dissimilar from those of other skiing-unrelated contexts, such as having been a guest in the same chalet [27,30], refuge/bar [30,31], or having participated in banquets [27] where the index case was present (asymptomatic or undiagnosed). In one study, the index case became symptomatic the same day of arrival and contagion had taken place earlier, when the case had attended workshops and meetings previous to traveling to the ski area [31]. The last study considered did not provide enough details to clarify the role played by ski areas [28].

The remaining three articles referring to the second and third epidemic phases (December 2020–February 2021) primarily focused on detecting and genotyping SARS-CoV-2 variants, including fewer details on the incidence and/or prevalence of SARS-CoV-2 infections among the reference population groups [34]. The first article, which analyzed wastewater samples (period June–December 2020) collected across various Swiss regions including a ski area, confirmed the presence of the B.1.1.7 variant strain (the so called “English” variant) in Switzerland earlier than previously thought; this suggests the Swiss case believed to have first contracted the variant and detected through human samples in fact occurred subsequently to previous unidentified cases [18]. The second article (dated 18 January 2021) referred to an intra-hospital cluster located in a skiing region (in a hospital in Garmisch-Partenkirchen). In this case, virus genotyping was ongoing at the time of article publication [19]. The third article, a seroprevalence study conducted in Cogne (Italy), reported a very low seroprevalence (i.e., <5%) in samples collected between March and May 2020 (including samples from the first and second epidemic waves), while a follow-up stage of the study focused on the potential reinfection of subjects who presented specific antibodies during the first stage [34]. This study is of particular interest for several reasons. Firstly, the reported seroprevalence rate is quite low when compared to other areas of Northern Italy over the same time period [2,6,7] and to similar reports included in the preprints we were able to retrieve. For instance, seroprevalence rates in other communities with access to ski resorts and in areas where a high incidence of COVID-19 was reported during the “first wave”, e.g., Blaine County and Ischgl, ranged between 22.9% and 42.4%, respectively [32,33]. Secondly, as the authors identified a reinfection rate of 5 out of 29 subjects during the second wave and only 11 out of 29 participants retaining protective levels of IgG-class antibodies, this study evidenced and addressed a more general and worrisome topic, i.e., the waning immunity elicited by natural SARS-CoV-2 infections. However, it should be stressed that the community of Cogne may not be comparable to other geographical areas as it is surrounded by four valleys and therefore substantially isolated from the nearby center of Aosta. Moreover, NPI were locally implemented very early during the first wave of the pandemic (March 2020) and more vigorously, particularly when compared to other ski areas across Northern Italy (e.g., the Lombardy and Trentino regions shut ski resorts only during the month of March 2020) [35], hereby somewhat anticipating the statutory interventions which followed at national level [34]. Therefore, it may be particularly difficult to ascertain the actual impact of ski resorts on the spreading of the pathogen. On the other hand, in the case study conducted on Blaine County, ski resort activities were halted belatedly in response to the NPI implemented by local authorities, and the nearby communities not directly involved in ski-related tourism had substantially lower rates than others where NPI were implemented over the same timeframe (19.4% vs. 34.9%) [32]. Not coincidentally, analyses of the Austrian outbreak equally pointed towards a mass spreading event that occurred in an après-ski bar, rather than in the ski area itself [31,33]. Lastly, the ECDC report assessed the general risk of COVID-19 transmission throughout the end-of-year festive season, only marginally referring to skiing activities. According to the ECDC, tourist resorts, especially during the winter season, are places where diffusion can easily take place because of public transportation, the attendance of gatherings, and a lower air exchange [20]. Characteristics of the included studies are synthetized in Table 1.

## 4. Discussion

The results of our rapid systematic review reveal that almost all retrieved studies were linked to the first epidemic wave (February–May 2020), with few articles assessing ski-related COVID-19 transmission during the second or third wave of the pandemic. The majority of included articles highlighted how the high diffusion and the strong impact of detected infection clusters was first and foremost related to the crowds and gatherings that took place without the application of restrictions or limitations. Nevertheless, it should be considered that at the beginning of the epidemic the use of masks was not recommended in the absence of symptoms and diagnostic tests were not routinely carried out, with the exception of symptomatic subjects with a travel history to Wuhan or close contacts of confirmed cases. In addition, at that time, there were no rapid tests available yet [36]. Only three articles focused on the second or third waves of the pandemic (December 2020–February 2021) and did not provide any detailed information regarding the specific involvement of ski areas in spreading the COVID-19 infection.

Based on the results of our rapid systematic review, there is currently no evidence of the specific role of ski areas in the transmission and spread of COVID-19. In fact, almost all retrieved articles report on studies conducted during the first epidemic wave, during which neither the epidemiological characteristics of the virus nor the impact of effective preventive measures were known. The only three identified articles assessing the link between recreational skiing and the pandemic during the second wave are a newspaper article, a study on 48 wastewater samples and a follow-up sub-analysis of the seroprevalence study performed in Cogne (Italy) during the “first wave” in early 2020. These recent news reports indicate that no increase in cases has been observed in the ski areas of countries where facilities are currently open, such as in Switzerland. On the other hand, available genotyping tests performed on leftover water samples collected from the ski resorts of Sun Valley and Ischgl stressed that activities after skiing rather than the ski areas themselves represent a potential setting at risk for spreading events. Lastly, the seroprevalence study in Cogne revealed how the early implementation of NPI largely reduced the spread of infection, particularly in settings were social and physical distancing are facilitated by the characteristics of the index community. However, there is a need to substantiate the current evidence base by systematically collecting data to confirm this observed trend. Moreover, it should be considered that skiing is an individual outdoor sport with limited interpersonal contact, therefore potentially not requiring strict preventive measures.

As highlighted by this rapid systematic review, none of the infected cases in ski areas directly developed in relation to the sport activity itself. In contrast, related activities such as living in the same hotels (sharing bedrooms), participating in gatherings, as well as eating at the same restaurants (particularly in absence of preventive measures) were responsible for the cases traced in the literature [37,38,39]. In light of this, it is important that health authorities focus their attention on implementing preventive and safety protocols, that take into account all potential risks, while also ensuring the reopening of ski areas at the same time. This would not only safeguard population health, but also guarantee the prosperity and economic survival of the mountain communities primarily involved in the skiing industry, while contributing indirectly to an entire country’s economy [40]. Such a safety protocol (summarized in Table 2) has been recently approved by the technical scientific committee advising the Italian government on the management of the COVID-19 pandemic.

The ECDC itself suggests that when restrictions are reduced, strategies that mitigate or contain the risk of infection should be put in place and that, based on potentially foreseeable sources that could cause a rise in cases, authorities should reorganize care and care systems to be able to accommodate for a potential increment in cases [20,41]. In addition, ECDC concludes by recommending that relaxation policies or the reinforcement of restrictions should be carefully weighed, seriously taking into account not only the epidemiological framework, but also the capacities of the health system, as well as the social, personal and economic impact of those measures.

Finally, there have been no explicit recommendations by the World Health Organization to halt sporting events of either professional or recreational nature. Instead, the WHO has created and disseminated a tool to determine the risk associated with the organization of such events. This tool also provides elements necessary to mitigate or reduce the risk of spreading the virus [25] and could be easily adapted and applied to guide policymakers in deciding whether to close or open ski areas.

Interestingly, despite the availability of the above-mentioned safety protocol issued by the Italian CTS, the included recommendations have never been adopted. In fact, several other governmental orders have impeded the reopening of ski areas for recreational purposes without geographical, epidemiological or logistic exemptions. In Italy, only professional ski activities subjected to an ad hoc safety protocol approved by the CTS have been permitted throughout the 2020 and 2021 winter season. The 2021 FIS Alpine World Ski Championships hosted in Cortina (Veneto region are a notable example. The competition took place in February 2021 in full accordance with the specific safety protocol and under the strict supervision of Local Public Health Authorities. The applied protocol estimated the risk level of the event through the above-mentioned WHO tool. More importantly, the protocol was the first to introduce the concept of “bubbles”. This system aimed at dividing the participants into homogeneous working groups (bubbles) in order to reduce contacts and the sharing of workspaces throughout the event. Consequently, athletes and team members were separated from media, event staff, and guests. Moreover, each group had to comply with general rules and bubble-specific roles. This facilitated the separation between groups, while also improving the tracking of any potential close contacts.

### Strengths and Limitations

Some strengths and limitations should be addressed before generalizing the results. Firstly, this was a rapid review, which, despite being systematic in nature, was limited to only four databases. Nevertheless, the assessment of four databases is in line with the minimum requirements (at least two) set by the PRISMA guidelines for systematic reviews. Secondly, we limited our search to articles published in English. However, since no articles were removed because of this language limitation, we are confident that our results are not affected by selection bias. Lastly, to this day, the literature concerning this topic is still relatively sparse, allowing us to only draw preliminary conclusions. Nevertheless, to the best of our knowledge, this is the first review assessing the role of skiing areas and related activities on COVID-19 spread, covering an extremely relevant topic not only from a Public Health perspective, but also for policymakers involved in strategic political decisions with a high impact on health, social and economic policy.

## 5. Conclusions

Despite the fact that several countries decided to postpone the reopening of ski areas, there is currently no robust evidence to support the association between COVID-19 spread and skiing, and no strong conclusions can be drawn at this point in time. Only a very small number of articles, mainly referring to the first epidemic wave, during which the epidemiological patterns of the pandemic were scarcely known and few or no NPI had yet been implemented, have been retrieved. This aspect is integral to our main results because it highlights a gap in knowledge that needs to be studied further. Moreover, the available evidence suggests that SARS-CoV-2 transmission in ski areas was not directly related to the sport activity itself and that, for all cases traced in literature, the setting was comparable to other contexts in which crowding can occur, such as staying in the same hotel/chalet or dining at the same restaurants without restrictions. Considering this and that skiing is an individual outdoor sport with limited interpersonal contact, it may be reasonable to consider the reopening of ski areas in compliance with strict rules, preventive and protective measures, such as those approved by the Italian Government and recently piloted in occasion of professional skiing competitions. At this stage, it seems coherent with the available scientific evidence to consider the possibility of allowing the utilization of ski areas in compliance with rigorous and widespread implementation of preventive measures such as social distancing, surgical masks, electronic ski-passes and potentially a health passport which admits all individuals who have been vaccinated or who can provide a negative rapid test result.

## Figures and Tables

**Table 1:** Characteristics of the included articles.

Author, Year [Reference]	Country	Study Period	Cluster Generated	Transmission
Aherfi, 2020 [27]	France (Alps)	8–15 March 2020 (Purin holidays)	Out of 50 subjects, 17 tested positive of which 16 from Marseille and 1 from Nice. Mean age 32 years, one pregnant woman, and 4 children.	In the hotel where families organized a week-long stay, sharing bedrooms, meals and participating in public events. The index case was not clearly identified.
Brandl, 2020 [28]	Germany	18 February–12 March 2020	Out of the first 110 pts detected in Germany, 11% had a skiing vacation in Austria or Italy.	No information provided.
Correa-Martinez, 2020 [29]	Austria (Ischgl)	9–16 March 2020	Out of 90 pts at University Hospital Munster (Germany), 36 tested positive and were in Ischgl, whereas other 9 pts had equivocal tests (61% male, aged 20–71 years). Norway reported 161 cases imported from Austria (no other details are provided). Iceland: travelers returning from Ischgl (no details info are provided).	Most of the German pts had visited apres-ski bars (on 7 March, an employee of an apres-ski bar tested positive, potentially the index case).
European Centre for Disease prevention and Control, 2020 [20]	European level	Not applicable	Not applicable.	Tourist locations, especially in the winter period, can be places where diffusion can take place more easily (use of public transport, crowding, less air exchange). The choices of easing or reinforcing restrictions should take into account the epidemiological framework, the capabilities of the health system, as well as the social, personal and economic impact of such measures.
Hodcroft, 2020 [30]	France (Contamnies-Monjoie; Haute-Savoie)	24 January 2020	A total of 11 positive cases were detected in UK and Spain starting from an index case (UK man).	A British man spent 2 days in Singapore, and before returning to the UK spent 4 days in Contamnies-Monjoie, sharing the same chalet with other 21 people from the UK (5 tested positive). Other 4 UK subjects tested positive, of which 3 were in the same skiing trip to France. The UK man had an unidentified contact (in the ski resort) with a Spanish man who became infected. The UK man had a yoga lesson in UK and infected another attendee.
Jahn, 2021 [18]	48 wastewater samples in Switzerland (Zurich, Lausanne and an alpine ski area)	8 July and 21 December 2020	Not applicable	In the ski area sample (21 December), 10 out of the 17 B.1.1.7 mutations and one of the 12 501.V2 mutations were detected. This suggests an earlier circulation of the B.1.1.7 variants compared to the previously believed first identified pt in Geneva (the 22 December).
Knabl L et al. 2020 [32]	Austria, Ischgl	21–27 April 2020	Cross sectional study on the entire population of Ischgl (No. = 1867); seroprevalence equals to 42.4%	Suspected role of an après-ski bar in the ski resort.
Kreidl, 2020 [31]	Austria (Tyrol; District of Imst, Kuhtai)	24–26 January 2020	A 33-year-old German woman became symptomatic (rhinitis, mild otitis, hyposmia and hypogeusia) the day of arrival in the Alpine resort.	The woman contracted the infection from a Chinese instructor who participated in several meetings and workshops in Starnberg, Bavaria.
McLaughlin C et al. 2020 [33]	USA, Idaho	8–9 April 2020	A series of 505 incident cases occurring in Blaine County (tot. Inhabitants, 23,089 population) during March-middle April 2020 (incidence rate, 2.9%); seroprevalence assessed on 917 volunteers; seroprevalence was higher (34.8% in Ketchum) in areas not directly involved in the ski resort, and lower in Sun Valley, where the ski resorts actually are (19.4%).	Tourist location, but the causes of the initial spreading of the outbreak remain unclear.
The Local, 2021 [19]	Germany (Garmisch-Partenkirchen Hospital in Bavaria)	18 January 2021	No information provided	52 patients and 21 employees had tested positive for SARS-CoV-2.
Truc F, Gervino G 2021 [34]	Italy, Cogne	3 March, 18 May, 2020	Cross-sectional study on the entire population of Cogne (No. = 1149); actual participants 857; seroprevalence equals to 3.4%.	Preventive effect of early implementation of non-pharmaceutical interventions (NPI), mainly represented by lockdown measures.

Pt = patients.

**Table 2:** Summary of proposed measures in the protocol for the safe access to ski areas approved by the Italian technical scientific committee (*Comitato tecnico scientifico*).

Area of Intervention	Proposed Measures
Transportation	Chairlifts to be used at 100% capacity and mandatory use of face masks. The capacity should be reduced to 50% in case of closed chairlifts. Gondola lifts/cable cars to be used at 50% capacity (in coherence with rules applied in public transportation) both for ascending and descending passengers. In case of exceptional or severe weather conditions, during which ski transportations are requested for descent, lifts may be used at full capacity for the required time only. Passengers should wear masks at all times.
Access to facilities	Limitations to number of daily, weekly, monthly passes for sale. Online booking systems, use of contactless ski cards. Separate entry and exit routes throughout facilities.
Catering	Take-away, service at tables, prebooking system for tables, tables outdoors.
Staff	Adapt work spaces to allow at least 1 m of distance between employees. Supply of protective equipment for staff (including gloves, surgical masks, FFP2 and 3, facial protection, goggles).
General protective measures	Appropriate COVID-19 surveillance, sanitation and ventilation of common areas, effective communication to inform customers and staff on existing rules/procedures.
Note: FFP: Filtering Facepiece	

## Data Availability

The data presented in this study are available on request from the corresponding author.
